# Health Behaviors of Childhood Cancer Survivors

**DOI:** 10.3390/children1030355

**Published:** 2014-10-22

**Authors:** Jennifer S. Ford, Marie Barnett, Rachel Werk

**Affiliations:** Memorial Sloan Kettering Cancer Center, Department of Psychiatry and Behavioral Sciences, 641 Lexington Avenue, 7th Floor, New York, NY 10022, USA; E-Mails: marie.barnett@gmail.com (M.B.); rswerk@ufl.edu (R.W.)

**Keywords:** childhood cancer survivors, health behaviors, pediatric cancer, alcohol use, tobacco use, exercise, diet, sun protection

## Abstract

There has been a dramatic increase in the number of childhood cancer survivors living to an old age due to improved cancer treatments. However, these survivors are at risk of numerous late effects as a result of their cancer therapy. Engaging in protective health behaviors and limiting health damaging behaviors are vitally important for these survivors given their increased risks. We reviewed the literature on childhood cancer survivors’ health behaviors by searching for published data and conference proceedings. We examine the prevalence of a variety of health behaviors among childhood cancer survivors, identify significant risk factors, and describe health behavior interventions for survivors.

## 1. Introduction

As a result of improved cancer treatments, there has been a dramatic increase in the number of young survivors who are living well into adulthood [1,2]. As a result of their cancer therapy, long-term survivors of childhood cancer are at risk for late complications (*i.e.*, late effects) ranging from relatively minor and easily treatable (e.g., underactive thyroid) to serious and occasionally fatal (e.g., second new cancer or early heart disease) conditions. As many as two-thirds of childhood cancer survivors are likely to experience at least one late effect, with approximately one-fourth of survivors experiencing a late effect that is severe or life threatening [3,4,5,6].

Many of these treatment-induced complications may not become apparent for decades after cancer treatment has completed. Common types of late-effects of childhood cancer include cardiopulmonary problems, endocrine disorders, musculoskeletal problems, neurocognitive and psychological problems, osteopenia or osteoporosis, and second malignancies [7,8,9,10,11,12,13,14,15,16,17,18]. Childhood cancer survivors have a 10-fold risk of developing an additional primary malignant neoplasm, a risk that continues into adult life [5,19]. In addition to late effects, childhood survivors are vulnerable to other chronic conditions akin to the general population. Therefore, it is imperative that survivors minimize preventable risk factors of cardiac, pulmonary, neoplastic, and other major diseases via positive health behaviors and lifestyle choices.

Lifestyle factors are known to influence an individual’s risk of disease in the general population [20,21,22,23]. Positive health behaviors are known to reduce the risk of cardiovascular disease, diabetes, cancer, and osteoporosis [24]. Engaging in a healthy lifestyle, by avoiding tobacco use, regularly exercising, having good nutritional habits, limiting alcohol use, and practicing sun protection is particularly crucial for these cancer survivors.

A cancer diagnosis during childhood or adolescence may have a unique and lasting impact on the development of lifelong health behaviors [25,26]. Smoking has been documented to begin and escalate throughout childhood and adolescence [27] and dietary and exercise habits may originate in childhood and are established more permanently during adolescence [28]. Survivors who participate in health damaging behaviors such as cigarette smoking may engage in multiple risk behaviors, including less health promoting behaviors [29,30]. As such, routine follow-up care for cancer survivors diagnosed during childhood and adolescence should aim to encourage the adoption of health-promoting behaviors and strongly counsel avoidance of health-damaging behaviors. This is especially crucial during a developmental period when normative experimentation with health-damaging behaviors and potential future dependence, pose a threat to the overall health and well-being for childhood cancer survivors [31].

A cancer diagnosis may also present an opportunity for a “teachable moment” to convey health behavior information and health risks with significant impact [32]. Among adults, several studies have demonstrated that a diagnosis of a life-threatening chronic illness motivates individuals to reduce health risks and engage in health-protective behaviors [33]. A cancer diagnosis during childhood could foster and motivate the adoption of a healthy lifestyle or childhood cancer survivors might engage in risky health behaviors at rates similar to their “healthy” peers, disregarding the additional risks due to their cancer history.

The goal of this article is to identify and critically evaluate the empirical literature published on childhood cancer survivors’ health behaviors. We will attempt to answer the following questions: (1) Do survivors of childhood cancer engage in more or less risky and positive health behaviors than peers? (2) What risk factors have been identified as significantly related to health behaviors among childhood cancer survivors? and (3) Are health behavior interventions for childhood cancer survivors effective? The wide range of included studies provides a broad picture of the literature.

## 2. Method

In preparing this article, a comprehensive literature search was performed to identify papers and books on childhood cancer survivors’ health behaviors following published methodological guidelines for review papers [34]. Relevant articles were identified by using PsychInfo, Medline, Cancerlit, and PubMed computerized databases of psychological and medical literature. Additional literature was identified from references in published papers and books. Unpublished work was excluded unless it appeared in conference proceedings.

Search parameters included studies published in English focused on individuals diagnosed with cancer during childhood, prior to the age of 21. The computer-based information search used the following key words: adolescent cancer, adolescent cancer survivor(s), alcohol use, cancer patient(s), cancer prevention, cancer survivor(s), childhood cancer, childhood cancer survivor(s), diet, exercise, health behavior(s), health promotion, health protection, lifestyle behavior(s), nutrition, pediatric cancer, pediatric cancer survivor(s), post-treatment, smoking, sun protection, and tobacco use. We did not include papers that focused on treatment or follow-up adherence as the sole health behavior of interest.

## 3. Results and Discussion

### 3.1. Health-Damaging Behaviors

The majority of the published studies focusing on childhood cancer survivors have focused on survivors’ tobacco use, with fewer studies examining alcohol and illicit drug use.

#### 3.1.1. Tobacco Use

##### 3.1.1.1. Prevalence

Several studies have reported that fewer cancer survivors smoke cigarettes compared to individuals in the general population [35,36,37,38,39,40,41,42,43,44], best friends [45], or siblings [36,46], however one study did demonstrate that survivors were more likely to be current smokers compared to non-cancer controls [47]. A majority of these studies focused on adult survivors of childhood cancers. Prevalence rates of tobacco use among childhood cancer survivors have ranged from 8% to 29% [35,46,48,49,50]. Additionally, survivors who do smoke have been found to smoke on average 1.5 cigarettes less a day compared to the general population [40], but have not been found to differ significantly from their siblings [29,51]. The lowest rates of tobacco use have been found among preadolescents and adolescents [48] and young adult cancer survivors are more likely to smoke compared to their older cancer-typed matched counterparts [52]. Quit rates are varied with some studies demonstrating survivors are not more likely to quit, compared to their siblings [46,48,53], while another study showed the opposite when compared to the general population [54]. Researchers have also described a trend toward increased tobacco use as cancer survivors reach late adolescence or young adulthood [46,55], increasing their risk for continued smoking [56]. Smoking rates may differ by cancer diagnosis as well [57,58].

When examining future intentions to use tobacco, younger survivors have reported low intentions to smoke in the future [37], while those with lower tobacco-related knowledge report greater future intentions to smoke [37], which is consistent with findings among healthy adolescents [59]. Self-reported intention to use tobacco has consistently been used as a proximal outcome measure in adolescent smoking research because prospective studies [54,60,61] have demonstrated that smoking intentions are a strong predictor of future smoking behavior.

##### 3.1.1.2. Risk Factors

Factors related to a greater likelihood of survivors using tobacco include being Caucasian [46], non-Black [35], or female when a close relative is a smoker [62]. However, this gender difference has not been consistently replicated in the literature. Overall, younger survivors, those of higher socioeconomic status, and those with higher educational achievement have been found to engage in less tobacco use [35,37,46,63,64,65], similar to the general population.

Current smoking and a higher amount of cigarette use among adult survivors of childhood cancers has also been associated with absence of radiotherapy treatment, those in manual occupations, those separated, widowed, or divorced, those with lower educational status, not currently in long-term regular follow-up medical care, history of attention deficits in childhood, and no smoking prohibitions at home and/or at work [40]. Some studies have also found a positive relationship between central nervous system treatment and risky health behaviors [66] while others have reported the opposite relationship between cranial radiation and tobacco initiation [35].

Several risk factors for continued smoking among adult survivors of childhood cancers include being younger than 14 years of age at smoking initiation, not having graduated high school, cranial radiation treatment, and being older at diagnosis [35].

Although preadolescent and adolescent survivors are moderately knowledgeable about tobacco-related health risks and perceive themselves as vulnerable to these risks, one study found that less than half accurately identified specific treatment-related health risks that could be exacerbated by tobacco use [50]. Those adolescents with lower knowledge scores reported greater intentions to use tobacco, consistent with findings among “healthy” adolescents [59]. Among survivors, perceived vulnerability is associated with smoking behavior [67,68]. Psychological distress has also been found to predict smoking [69], and smoking may be related to psychological morbidity including anxiety and depression [70].

#### 3.1.2. Alcohol Use

##### 3.1.2.1. Prevalence

Several studies have assessed alcohol use among adolescent and adult survivors of childhood cancers and have found prevalence rates ranging from 8% to 84% [36,48,71]. This wide range may be due to the varying methods of assessment and definitions of alcohol use/abuse. There are fairly low rates of risky drinking among survivors compared to controls [41,42,44,72,73], however up to 25% of survivors report binge drinking [38]. Lown and colleagues examined this more extensively and found that when compared to siblings, survivors were less likely to engage in risky or heavy drinking, however they were more likely to be current drinkers [74]. Other studies challenge this assertion, finding either no difference between alcohol use between survivors and case controls [56,71], or finding that survivors more often consumed alcohol than controls [75,76].

##### 3.1.2.2. Risk Factors

Most studies have found that younger individuals, those of higher socioeconomic status, black survivors, and those with higher educational achievement have been found to engage in less problematic alcohol use [35,37,46,63,64,65,77].

Heavy drinking among survivors has been found among males, between 18 and 21 years of age, do not have a college/university education, and initiate drinking before age 14 [74]. Among Hodgkin’s lymphoma survivors, a history of relapse predicted both moderate and heavy alcohol use [65]. Heavy drinking among survivors has been related to psychological distress [69], including symptoms of depression, somatization, and anxiety [74].

#### 3.1.3. Illicit Drug Use

##### 3.1.3.1. Prevalence

Many studies demonstrate that survivors, no matter what age, are less likely to use illicit drugs than the general population [36,45,71,78,79,80], with only a few studies showing no differences between the rates of cancer survivors’ drug use with sibling and general population controls [81].

Among teenage cancer survivors, illicit drug use rates have been reported between 8% and 16%, with higher rates either for males or for marijuana use [79]. Not only has illegal drug use been reported as lower among survivors, but also rates of experimentation with these substances are lower among survivors compared to peer controls [71]. Additionally, survivors have been found to experiment with fewer types of drugs [71]. For example, within a cohort of young adult AML survivors, the most common substances used were marijuana, tobacco, and alcohol [82]. Those who used cocaine, heroin, or methamphetamine comprised less than 10% of that survivor group [82]. Another consideration is how drug use is defined. In fact, one study showed that survivors 13–17 years old were more likely to use pain relievers for non-medical reasons but less likely to use cannabis [78].

##### 3.1.3.2. Risk Factors

Several risk factors for current illicit drug use among adolescent cancer survivors include older age, lower resiliency to peer influences, and drug use among friends and household members [83]. Risk factors of lifetime drug use among this cohort include executive functioning deficits, a history of current school problems [83], and poorer decision-making skills [79].

### 3.2. Health Damaging Behaviors: Interventions

Due to their health risks and prevalence of health damaging behaviors among childhood cancer survivors, successful cost-effective health behavior interventions are vital [84]. To date, intervention strategies have included informational booklets [85] and other print formats [8,86], telephone counseling [61,84,87,88,89], peer counseling groups [84], and e-Health components which include video gaming, individual messaging, social networking [90], web based formats [91,92], and educational videos [61]. The majority of risky health behavior interventions to date have focused on tobacco use of adult survivors of childhood cancers. Emmons and colleagues assessed the effectiveness of various types of smoking cessation interventions and found that when adult survivors of childhood cancers who smoke participated in a peer counseling program, rather than a self-help group, they were twice as likely to quit smoking [84] and their long-term quit rates were higher [89]. Smokers who received nicotine replacement therapy patches as a part of a smoking cessation intervention were more likely to make a 24-hour quit attempt than those who only received counseling calls and materials [88].

Among “healthy” adolescents, decision-making skills are among the important determinants of behavioral change [93]. This has led to several interventions focused on decision-making programs for risk-reduction among childhood cancer survivors [94,95,96]. Cox and colleagues reported that in their behavior-change intervention program, conducted with adolescent survivors, that perceptions about the need to change behaviors increased smoking abstinence maintained for 1 year [94].

It is noteworthy to mention that only 42% of survivors are asked about their smoking behaviors by their health care providers [97,98], and only 55% of childhood cancer survivors who smoked reported receiving advice to quit smoking from their health care provider [86]. Nathan and colleagues found that only approximately 18% of survivors surveyed report that their medical appointments related to their previous cancers include discussion or counseling of the risks associated with their prior diagnoses [99,100], which is relevant and important when thinking about risky health behaviors for this cohort. Health care professionals must become more involved in the risky health behavior prevention, education, and intervention process [95,101,102].

### 3.3. Health Protective Behaviors

Survivors more often engage in positive health promoting behavior changes [103] after a cancer diagnosis. Overall younger individuals, those of higher socioeconomic status, with greater adult social support, and with higher educational achievement have been found to engage in greater protective health behaviors, namely healthy diet and physical activity [35,37,46,63,64].

#### 3.3.1. Physical Activity

##### 3.3.1.1. Prevalence

Physical activity has numerous beneficial effects on physical and mental health among childhood cancer survivors [104]. Despite these benefits, adolescent and adult survivors of childhood cancers have been found to be less physically active than siblings or age- and gender-matched population samples [105,106]. Up to 52% of childhood cancer survivors do not meet guidelines for weekly-recommended physical activity [57]. For example, Mulhern and colleagues reported that 6% of preadolescents-adolescents (11–17 years of age) and 17% of their young adults (18–30 years of age) were sedentary and exercised less than one hour a week [48].

There have been differences in physical activity levels reported between survivors of different cancer diagnoses [57,105,107,108], with lower levels of activity reported for survivors of neuroblastoma, soft tissue sarcomas, germ cell tumors, retinoblastoma, bone tumors and CNS tumors [108]. A majority (65%) of CNS tumors or lymphoma survivors do not meet national recommendations for physical activity [109,110]. Interestingly, physical activity levels and sedentary behavior do not differ between overweight and normal weight survivors [111,112].

##### 3.3.1.2. Risk Factors

There are several barriers to physical activity among childhood survivors, including being tired (57%), busy (53%), and not having a gym membership (48%) [113]. Physical limitations and participation constraints often hinder activity among survivors compared to healthy controls [114]. Family and peer support for physical activity is significantly related to survivor physical activity levels [115].

Cranial radiation or amputation, female gender, black race, older age, lower educational attainment, being underweight or obese, currently smoking, and depression are all associated with an inactive lifestyle among childhood cancer survivors [105]. Social withdrawal, antidepressant use, stress and low self-esteem among survivors of childhood cancer have also been associated with physical inactivity [116].

#### 3.3.2. Diet/Nutrition

##### 3.3.2.1. Prevalence

Reported rates of good dietary habits among survivors, range from 40% to 70% [48,50,96]. Studies report less than 25% of survivors eat balanced meals, similar to what is noted for non-cancer peers [48] and siblings [104]; however, other studies report that rates of survivors’ adherence to dietary guidelines are lower than those from an age-matched non-cancer population [42].

More specifically, most adolescent and adult survivors of childhood cancers do not meet guidelines for fruit and vegetable consumption, calcium intake, and calories from fat consumption [57,107]. Survivors also do not meet requirements for folate, calcium and iron intake and report dietary sodium and added sugar intake considerably in excess of recommendations [117].

##### 3.3.2.2. Risk Factors

Higher body fat and prior cranial irradiation has been associated with poorer diet [118]. Survivors with poorer parental relationships were significantly more likely to consume high-fat diets [119].

#### 3.3.3. Sun Protection

##### 3.3.3.1. Prevalence

Skin cancer is one of the most common secondary neoplasms among childhood cancer survivors with 37% diagnosed with a non-melanoma skin cancer and nearly 4% having melanoma [120]. Protecting one’s skin from the sun’s ultraviolet rays can reduce risk of skin cancer and a majority (64%) of adolescent cancer survivors reported engaging in the recommended sun protection [96] with similar patterns of sunscreen use among survivors and siblings [121]. Survivors were also significantly less likely to report having sunbathed in the previous year or artificially tanned than siblings [121]. However, more than a quarter (29%) of young adult survivors reported low sun protection adherence during sunbathing [122].

##### 3.3.3.2. Risk Factors

Significant factors for regular sunscreen use for survivors include female gender, lighter skin complexion, previous skin cancer screening, skin prone to sunburns when unprotected, and having had radiation exposure in the past year [121].

### 3.4. Health Protective Behaviors: Interventions

Evidence suggests that successful interventions should target multiple health protective behaviors (e.g., physical inactivity and diet). Compared with interventions focused on weight control, smoking cessation, or improving self-esteem, there is a high survivor interest consistently found for health promotion interventions aimed at improved physical activity and eating healthy [57,107,109,123]. Interventions focusing on improving physical activity result not only in increased exercise but also reduced fatigue [124] and improved cardiometabolic risk factor status [125].

Several technology driven interventions, utilizing Facebook, social networking sites and internet-based interventions, have been successful in increasing exercise and healthy diet [126,127]. Adherence to a Mediterranean diet among adult survivors of childhood ALL has shown to be associated with better metabolic and anthropometric parameters [128]. One pilot study to evaluate a mentored vegetable gardening intervention for cancer survivors found it to be both feasible and acceptable, demonstrating both self-reported and objective improvements in diet [129]. There is one successful and feasible intervention targeting sun safety practices for survivors that focused on using sunscreen and reapplying sunscreen regularly among adolescent survivors of childhood cancer [130].

### 3.5. Discussion

Greater health behavior research focused on childhood cancer survivors, with special attention paid to designing successful and cost-effective interventions are needed. To date, much of the research has either been solely descriptive in nature or been focused on tobacco use and smoking cessation interventions for childhood cancer survivors. As a result, there is a dearth of intervention studies focused on alcohol and drug use, diet and healthy nutrition, physical activity, and sun protection. The existing research also has some significant limitations including the fact that many of the aforementioned studies were conducted with small sample sizes, potentially limiting their conclusions and generalizability. Another limitation of the prior research important to note is that health behaviors were often not assessed using standardized methodologies and/or were asked utilizing only one or two questions, potentially diminishing the reliability and validity of the reported findings. This may explain, in part, the wide ranges of health behavior prevalence reported throughout the literature.

Another consideration to take into account when interpreting the published literature on health behaviors of childhood cancer survivors is that survivors in these studies were diagnosed with cancer at considerably varied age ranges, from birth to late adolescence/young adulthood. It is possible that individuals diagnosed at younger ages may be less knowledgeable about their diagnosis, potential late-effects, or the importance of maintaining a healthy lifestyle. It is also possible that these survivors were not cognitively developed enough to integrate their cancer experience into their health identity and subsequent health behaviors. One final shortcoming of these studies is that no study examined the full range of health behaviors directly relevant to cancer survivors’ increased health risks (e.g., nutrition, exercise, sun protection) within a single cohort.

There are several health behavior theories that have been utilized to identify risk factors and design health behavior interventions, including the Health Belief Model, the Stages of Change (Transtheoretical) Model, the Theory of Planned Behavior and the Precaution Adoption Process Model (e.g., [131]). Utilizing theory can help provide a systematic way to advance our understanding of childhood cancer survivors’ health behaviors can provide targets of focus to utilize for our interventions. However, many studies do not examine the relevant factors specific to childhood cancer survivors in a meaningful way; therefore, we cannot conclude from much of the prior research whether existing health behavior interventions for a non-cancer sample would be just as effective.

Given these short comings in the prior literature we make several recommendations in order for the field to advance. Studies should focus on the differences in risk factors and active intervention targets for a healthy sample as compared to a sample of childhood cancer survivors. Then, it would be possible to conclude what, if anything, about survivors’ cancer history and experience is relevant to their health behaviors and how best to target health behavior change. If there are no significant differences, then researchers and clinicians could just implement proven and successful health behavior interventions for survivors in the immediate future. Additionally, research should focus on a more full range of health behaviors and investigate the interplay between them in order to best understand the relationship between each, and to best understand ways to implement multiple health behavior interventions for survivors of childhood cancers. Lastly, the field would be advanced from an examination of health behavior theory in order to best assess the most appropriate theories for childhood cancer survivors and/or the creation and validation of new theories that would be more relevant to the adoption and maintenance of health behaviors for this high-risk group of individuals.

Because survivors are at higher risk of late effects given their cancer history, even studies that demonstrate low to moderate levels of health-damaging behaviors are alarming. In order to identify high-risk subgroups of survivors, we need further research to better identify significant risk factors of health behaviors to develop effective interventions.

## 4. Conclusions

In conclusion, there is a large body of literature exploring the initiation and adoption of health behaviors among “healthy” adolescents and young adults; however, the survivorship literature has not fully utilized these findings. Many of these studies were a-theoretical, a surprising finding given the breadth of theory-driven health behavior research on adolescents in the general population. By identifying psychosocial factors related to the adoption of health behaviors, we can begin to identify the high-risk subgroups of survivors who need targeted interventions [132].

### Future Directions

Although we have gathered a multitude of conclusions within the literature, there exists much disagreement between findings. Future research should target these inconsistencies so as to better define relationships and predictors of health damaging and health promoting behaviors among cancer survivors. Future efforts should also focus on developing successful interventions among cancer survivors to decrease health-damaging behaviors and increase health-promoting behaviors. Interventions should focus not only on the short-term outcomes of interest, but whether they result in long-term behavior change.
